# Plant Innate Immunity Induced by Flagellin Suppresses the Hypersensitive Response in Non-Host Plants Elicited by *Pseudomonas syringae* pv. *averrhoi*

**DOI:** 10.1371/journal.pone.0041056

**Published:** 2012-07-23

**Authors:** Chia-Fong Wei, Shih-Tien Hsu, Wen-Ling Deng, Yu-Der Wen, Hsiou-Chen Huang

**Affiliations:** 1 Graduate Institute of Biotechnology, National Chung Hsing University, Taichung, Taiwan; 2 Department of Plant Pathology, National Chung Hsing University, Taichung, Taiwan; 3 Department of Biology, National Changhua University of Education, Changhua, Taiwan; University of Wisconsin-Milwaukee, United States of America

## Abstract

A new pathogen, *Pseudomonas syringae* pv. *averrhoi* (Pav), which causes bacterial spot disease on carambola was identified in Taiwan in 1997. Many strains of this pathovar have been isolated from different locations and several varieties of hosts. Some of these strains, such as HL1, are nonmotile and elicit a strong hypersensitive response (HR) in nonhost tobacco leaves, while other strains, such as PA5, are motile and elicit a weak HR. Based on the image from a transmission electron microscope, the results showed that HL1 is flagellum-deficient and PA5 has normal flagella. Here we cloned and analyzed the *fliC* gene and glycosylation island from Pav HL1 and PA5. The amino acid sequences of FliC from HL1 and PA5 are identical to *P. s.* pvs. *tabaci* (Pta), *glycinea* and *phaseolicola* and share very high similarity with other pathovars of *P. syringae*. In contrast to the flagellin mutant PtaΔ*fliC*, PA5Δ*fliC* grows as well as wild type in the host plant, but it elicits stronger HR than wild type does in non-host plants. Furthermore, the purified Pav flagellin, but not the divergent flagellin from *Agrobacterium tumefaciens*, is able to impair the HR induced by PA5Δ*fliC*. PA5Δ*fgt1* possessing nonglycosylated flagella behaved as its wild type in both bacterial growth in host and HR elicitation. Flagellin was infiltrated into tobacco leaves either simultaneously with flagellum-deficient HL1 or prior to the inoculation of wild type HL1, and both treatments impaired the HR induced by HL1. Moreover, the HR elicited by PA5 and PA5Δ*fliC* was enhanced by the addition of cycloheximide, suggesting that the flagellin is one of the PAMPs (pathogen-associated molecular patterns) contributed to induce the PAMP-triggered immunity (PTI). Taken together, the results shown in this study reveal that flagellin in Pav is capable of suppressing HR via PTI induction during an incompatible interaction.

## Introduction

Plants utilize a refined network of defense mechanisms to protect themselves from the invasion of microorganism. Thus a successful bacterial pathogen has to overcome at least two levels of plant defense for its survival. The first level is a basal resistance which is induced by perception of microbial-associated molecular patterns or pathogen-associated molecular patterns (MAMPs or PAMPs) and thus the defense is also named PAMP-triggered immunity (PTI) [1]. Bacterial PAMPs include flagellin, cold-shock protein, elongation factor Tu (EF-Tu), and peptidoglycan [2] which are recognized by pattern recognition receptor (PRR)-like kinases [3]. Phytobacteria overcome this defense by injecting type III effectors (T3Es) via type III secretion system (T3SS) to suppress PTI. However, some plants have evolved a second level of defense by expressing resistance (R) proteins to recognize some of these effectors, thereby induce effector-triggered immunity (ETI) [4]. The common plant immune responses such as an oxidative burst, hormonal changes, and transcriptional reprogramming are triggered during PTI and ETI [2], [5], revealing that the profiles of those defense genes expressed in PTI and ETI are overlapped [6] and share some common signaling networks [7]. Compared to PTI, immune responses induced during ETI are more prolonged and much sturdier [8], [9]. The typical phenomenon of ETI has been distinguished from that of PTI by eliciting the localized programmed cell death termed hypersensitive response (HR) [4]. It has been observed that HR elicited by an ETI-inducing bacterial strain could be inhibited in the inoculated area where was pretreated with a PTI inducer, before the induction of HR was completed [10]–[12]. The HR inhibition by a PTI inducer refers to restriction of T3E injection and thus a successful pathogen ought to have active T3Es to overcome the restriction [13].

*Pseudomonas syringae* pv. *tomato* (Pto) flagellin is a well-characterized PAMP and is recognized by FLS2 in *Arabidopsis thaliana* [2]. The perception of flagellin occurs by recognition of the most conserved domain in its N terminus, represented by the peptide flg22 [14]. The flagellin and flg22 have been reported to induce PTI in tomato and *Arabidopsis* [14]–[16]. Interestingly, a flagellin-defective mutant (Δ*fliC*) of *P. syringae* pv. *tabaci* (Pta) 6605 lost the ability of causing the HR in nonhost tomato plants and reduced virulence for host tobacco plants [17]. The purified flagellin from *P. syringae* pv. *glycinea* (Pgl) race 4 but not from Pta 6605 triggers defense responses in tobacco although the sequences of the flagellin from both bacterial strains are identical [18]. And results obtained from the same research group suggest that the glycosylation of flagellin is responsible for the difference [19], [20]. The genes, *fgt1* and *fgt2*, of Pta required for flagellin glycosylation termed glycosylation island were located in the upstream of *fliC* gene, and also the glycosylation contributes to evasion of host tobacco plant surveillance system [20]–[22].

A new pathogen *P. syringae* pv. *averrhoi* (Pav) which causes bacterial spot disease in carambola (*Averrhoa carambola* L.) was reported in Taiwan [23]. Over 50 strains of Pav, such as HL1, HL2, HL9 and PA5, were isolated from different varieties of carambola plants in different locations of Taiwan area. Based on the preliminary analyses (Table 1), we observed that the strain HL1 elicits a stronger HR on nonhost tobacco leaves and is non-motile on soft nutrient gelatin agar (NGA) plate, whereas PA5 possesses motility and elicits a weak HR in the non-host tobacco plant. Both HL1 and PA5 cause typical leaf spot disease in the host plant carambola (i.e starfruit tree), suggesting that the motility is not a critical factor for virulence. Previous studies have demonstrated that motility apparently increases the infectivity of *P*. *syringae* pv. *phaseolicola* (Pph) in bean leaves [24] and of Pgl in soybean leaves [25]. Non-motile mutants of *Ralsotonia solanaceaum* are significantly reduced in virulence on tomato plants, indicating that motility plays an important ecological role in plant-microbe interactions [26]. A polar flagellum of *Acidovorax citrulli* was demonstrated to be required for full virulence before and after penetrating the tissue of melon hosts [27]. Nevertheless, flagellin is not required for the virulence of Pto DC3000 on the host *Arabidopsis* plant when pathogen was infiltrated into leaves [16]. Those variable observations led us to investigate the role of flagellum in the Pav-plant interactions.

**Table 1:** The motility and ability of hypersensitive response (HR) elicitation of *P*. *syringae* pv. *averrhoi* strains.

Strains	Sources(cultivar)^a^	Motility^b^	Moving scale(mm) c	HR^d^
HL1	Fruit (Unknown)	–e	–	<12 h^f^
HL2	Leaf (Unknown)	++	7.0 (±0.41)	>48 h
HL3	Leaf (Unknown)	++	7.5 (±0.64)	>48 h
HL6	Leaf (Unknown)	++	9.8 (±0.25)	>48 h
HL7	Leaf (Unknown)	+	2.5 (±0.50)	24h∼48 h
HL9	Leaf (Unknown)	++	9.5 (±1.04)	24h∼48 h
PA1	Leaf (Malaysia)	++	15.3 (±0.25)	>48 h
PA2	Leaf (Malaysia)	–	–	<12 h
PA5	Fruit (Malaysia)	++	15.8 (±0.48)	>48 h
PA6	Fruit (Malaysia)	+	3.5 (±0.29)	12h∼24 h
PA13	Leaf (Tsen Tsway)	++	15.3 (±0.63)	12h∼24 h
PA17	Leaf (Tsen Tsway)	++	14.8 (±0.48)	24h∼48 h

a Data were adapted from the report by Lin et al [23].

b This swimming assay was performed on 0.3% soft nutrient gelatin agar (NGA) plate.

c The scale shown with ±standard error is the mean diameter of swimming area and is calculated on triplicates.

d The bacteria were inoculated in tobacco leaves at 10^8^ cfu/ml.

e Symbol -, + and ++ denote respectively that bacteria are non-motile, swimming slowly and swimming well.

f The time represents the appearance of necrosis symptom and was recorded within 48 h.

In this study, we observed that PavHL1 is a flagellum-defective strain by using transmission electron microscope (TEM). We also generated *fliC* mutants of Pav strains PA5, HL2, and HL9 and compared the ability of HR elicitation between the *fliC* mutants and their wild types on tobacco leaves. In contrast to the Pta flagella [18], Pav flagella are not involved in host-range determination but possess negative effects on the HR elicitation. That is, the motile strains of Pav cause the delayed HR, which resulted from the suppression by the enhanced PTI induced by flagellin at the early step of an incompatible interaction. Moreover, this activity is independent on the glycosylation of flagellin.

## Results

### Cloning and Sequencing Analysis of Flagellin Gene and Glycosylation Island from *P. Syringae* Pv. *Averhoi* HL1 and PA5

**Figure 1:**
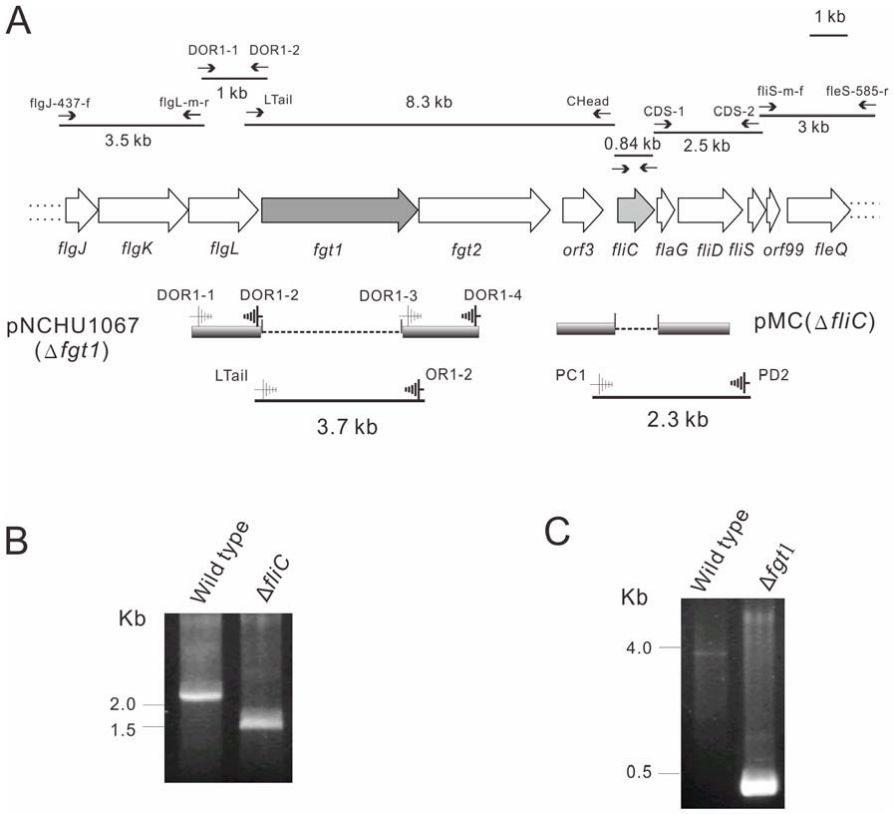
Cloning of flagellum gene cluster and glycosylation island and construction of *fliC* and *fgt1* mutants. (A) Organization of partial flagellum gene cluster and glycosylation island. The black lines represent the PCR fragments amplified by indicated primer pairs (see Table S1) as shown with arrows above the fragments. The constructs used to delete *fliC* and *fgt1* genes are aligned below the gene structure. (B) *fliC* mutant was screened by PCR using primers PC1 and PD2 as indicated in Figure 1A. (C) *fgt1* mutant was screened by PCR using primers prLTail and prOR1–2 as indicated in Figure 1A.

Since the amino acid sequences of flagellin from different bacterial species are very conserved in their N-terminal and C-terminal regions [28], we used the primer pair, prfliC-f and prfliC-r (Table S1) designed according to the conserved 5′- and 3′-terminal sequences of *fliC* genes from a few pathovars of *P. syringae*, to amplify *fliC* gene from HL1 and PA5, and a 0.84 kb PCR product was subsequently cloned into pGEM-T easy vector (Promega) and sequenced. Following the same strategy to search for the conserved sequence among *P*. *syringae* pathovars, an 8.3 kb glycosylation island was amplified from chromosomal DNAs of PA5 and HL1 as templates by Long-PCR using a primer pair (prCHead and prLTail) and was cloned into pCR-XL-TOPO (Invitrogen). Meanwhile, the genes flanking *fliC* and glycosylation island were cloned by PCR with primer pairs: prfliS-m-f/prfleS-585-r for generating 3 kb; prCDS-1/prCDS-2 for 2.5 kb; prDOR1-1/prDOR1-2 for 1 kb; and prflgJ-437-f/prflgL-m-r for 3.5 kb fragment respectively (Figure 1A). Sequencing analysis of those DNA fragments reveals those gene products including FlgJKL, FlaG, FliDS and FleQ are involved in flagellar biogenesis (Figure 1A) [29] and the gene organization is the same as to other pathovars of *P. syringae* [20]. The sequences of flagellum related genes and glycosylation islands from HL1 and PA5 are identical and share high similarity (80–100%) with other pathovars of *P. syringae* (Table S2). Moreover, the amino acid sequences of FliC from HL1 and PA5 are 100% identical to Pta, Pph, and Pgl (Table S2).

### HL1 is non-motile and Flagellum-deficient, but PA5 Possesses Motility and Flagella

**Figure 2:**
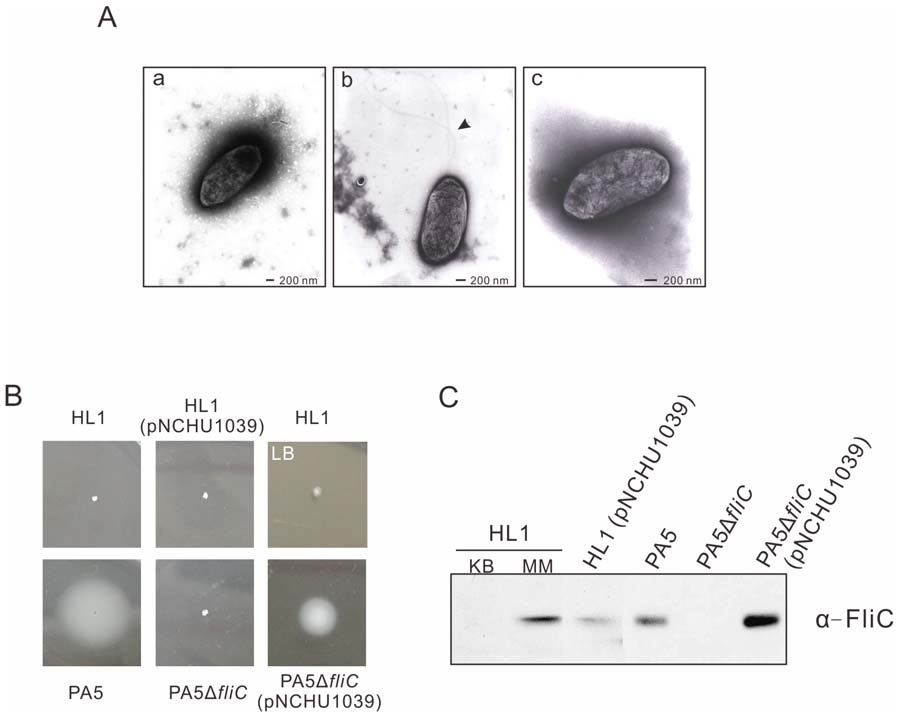
Flagellar expression and motility of *P. syringae* pv. *averrhoi* strains. (A) TEM images of *P. syringae* pv. *averrhoi* strains HL1 (a), PA5 (b), PA5Δ*fliC* (c) grown for 24 h on KB plates, deposited on TEM grids. Bars represent 200 nm and the arrow indicates the flagella only presented in wild type PA5. (B) Swimming assay. Bacterial strains were incubated for 2 days at 23°C on soft agar of *hrp* minimal medium or LB containing 10 mM MgCl_2_ plate (as indicated as LB). (C) Immunoblot analysis of flagellin expression in HL1, PA5, *fliC* mutant and complemented strain was detected by anti-FliC antiserum. Except for the flagellin of HL1 induced in *hrp* minimal medium (MM), the others were prepared from cultures in KB medium. pNCHU1039: pBBR1MCS5 carrying *fliC*.

In the preliminary analysis, we found that HL1 is not motile on NGA soft agar but PA5 is. Based on the observation under TEM, the images showed that the flagellum is not synthesized in HL1 (Figure 2A-a), but PA5 possesses at least two intact flagella (Figure 2A–b). To further determine the motility of HL1 and PA5 on various media, we evaluated the swimming motility on 0.3% soft agar plates of *hrp*MM and LB-MgCl_2_, respectively. The results showed that HL1 is indeed non-motile on both types of media (Figure 2B), whereas PA5 swims very well on *hrp*MM (Figure 2B), NGA, and LB soft agar plate (data not shown). According to the immunoblot assay, FliC protein produced within HL1 cells was barely detected in a rich medium (KB) compared to a large amount of FliC induced in the *hrp*MM (Figure 2C). Nevertheless, the secretion of FliC in HL1 was not detected in these medium, either (data not shown). Surprisingly, as the extra copy of *fliC* carried in pNCHU1039 was expressed in HL1 (Figure 2C), the motility was not recovered (Figure 2B), suggesting that there are unidentified factors involved in the regulation of *fliC* expression in *hrp*MM and the loss of flagellum biogenesis in HL1.

### *fliC* Mutant of Pav Causes a Stronger HR than its Wild Type does

In contrast to the result showing a loss of HR-eliciting on its nonhost plants in *fliC* mutant of Pta 6605 [17], the aflagellar HL1 still causes a strong HR on non-host plants including tobacco and tomato and the flagellar PA5 elicits a weak HR (Table 1). It led us to investigate whether the flagella have negative effects on the HR elicitation by Pav. To make flagellin-deficient PA5, the *fliC* mutant was generated as depicted in Figure 1A and 1B. PA5Δ*fliC* is aflagella observed from TEM (Figure 2A–c) and is non-motile on the *hrp*MM soft agar (Figure 2B). No flagellin production in PA5Δ*fliC* was further confirmed by an immunoblot probed with FliC polyclonal antibody (Figure 2C), and FliC production/motility was restored by complementing PA5Δ*fliC* with pNCHU1039 containing *fliC* (Figure 2B and 2C). Taken together, data reveal that *fliC* is the only gene coding for flagellin to assemble flagella in Pav.

**Figure 3:**
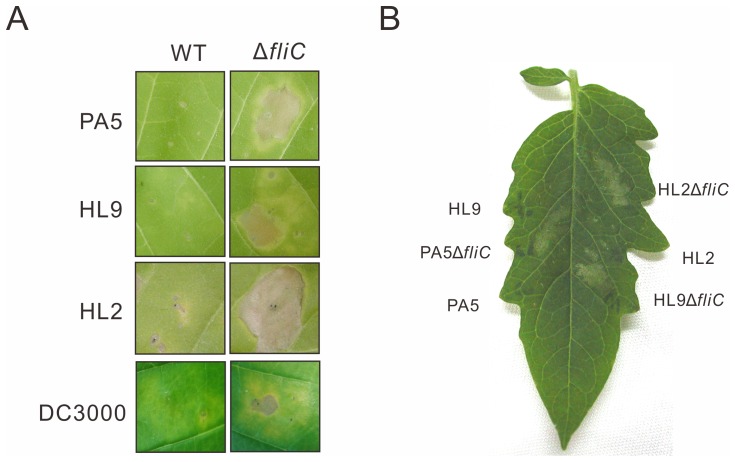
*fliC* mutants of Pav elicited stronger HR in nonhost tobacco and tomato leaves. (A) Tobacco leaves (*Nicotiana tabacum* L. cv. Van-Hicks) were infiltrated with 2.5×10^7^ cfu/ml of *P. syringae* pv. *averrhoi* strains PA5, HL2, and HL9 and 2×10^5^ cfu/ml of *P. s.* pv. *tomato* DC3000 and photographed at 6 days after inoculation. (B) Tomato leaf (*Solanum lycopersicum* cv. Moneymaker) was infiltrated with the indicated strains at a concentration of 1×10^7 ^cfu/ml and photographed at 1 day after inoculation.

In the HR assay, the tobacco leaves (*Nicotiana tabacum* L. cv. Van-Hicks) were inoculated with PA5 and PA5Δ*fliC* at the concentration of 1×10^8 ^cfu/ml, the HR-associated necrosis elicited by PA5Δ*fliC* emerged faster than that by PA5 (data not shown). Further, we used a lower inoculum of 2.5×10^7^ cfu/ml, closed to the threshold required for PA5 to elicit a macroscopic HR in the tobacco leaves, to determine the intensity of HR elicitation. PA5Δ*fliC* caused necrosis lesions within 2 days after inoculation, but the wild type didn’t until 6 days after inoculation (Figure 3A). Other motile strains such as Pav HL2 and HL9 which elicit the weak HR in tobacco were also investigated. The *fliC* mutants of HL2 and HL9 were constructed by the same strategy. Consistent with the PA5Δ*fliC*, the *fliC* knockout mutants of HL2 and HL9 elicited the stronger HR in tobacco leaves than their wild type strains did (Figure 3A). These strains were also inoculated into other nonhosts, tomato (cv. Moneymaker) (Figure 3B) and *N*. *benthamiana* (data not shown) at 1×10^7^ cfu/ml. As expected, the *fliC* mutants caused stronger HR than wild type in these non-host plants. Furthermore, the *fliC* mutant of Pto DC3000, a well studied pathovar, also causes stronger HR than wild type does (Figure 3A), suggesting that this phenomenon is not unique for pathovar *averrhoi*.

**Figure 4:**
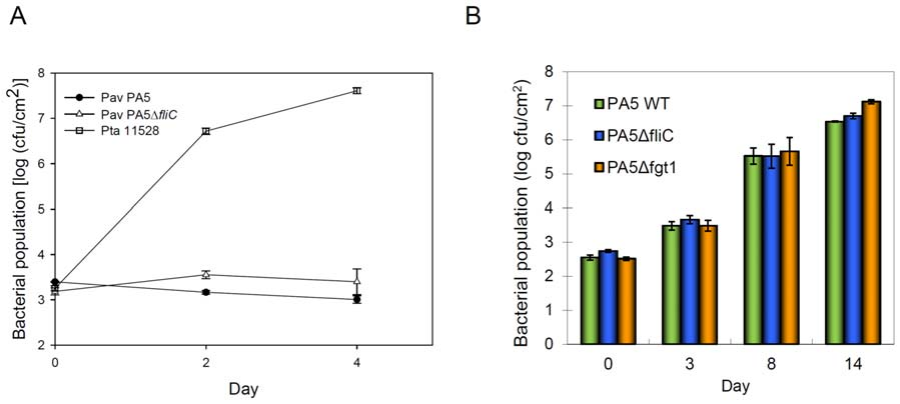
Bacterial growth in nonhost tobacco and host starfruit leaves. (A) Tobacco leaves were infiltrated with 1×10^5^ cfu/ml of indicated strains. *P. syringae* pv. *tabaci* 11528 is virulent to tobacco and was served as a host strain for a positive control. (B) Starfruit leaves were infiltrated with1×10^5^ cfu/ml of indicated strains. The populations were measured from three 0.6-cm-diameter leaf discs at indicated days post inoculation. Error bars indicate the standard error of populations measured from three leaf discs from each of two plants. The experiment was repeated three times with similar results.

To rule out the necrosis caused by *fliC* mutants of Pav is disease-like symptom, we determined the bacterial growth in tobacco. PA5, PA5Δ*fliC* and a compatible strain Pta 11528 were inoculated into tobacco leaves by syringe-infiltration at the concentration of 1x10^5^ cfu/ml. The results showed that the growth of PA5 and PA5Δ*fliC* were retarded in tobacco compared to Pta 11528 that grew to 10^7 ^cfu/cm^2^ at 4 days and caused disease symptoms at 6 days after inoculation (Figure 4A). To determine the virulence of *fliC* mutant in carambola, we inoculated PA5Δ*fliC* and its wild type into carambola leaves at the concentration of 1×10^5^ cfu/ml by syringe-infiltration. PA5Δ*fliC* grew up to a comparable level with wild type (Figure 4B) suggesting that the Pav flagellum does not act as a virulence factor when the pathogen was infiltrated into the intercellular space of leaves.

### Flagellin Impairs the HR on Tobacco Leaves Induced by PA5Δ*fliC*

**Figure 5:**
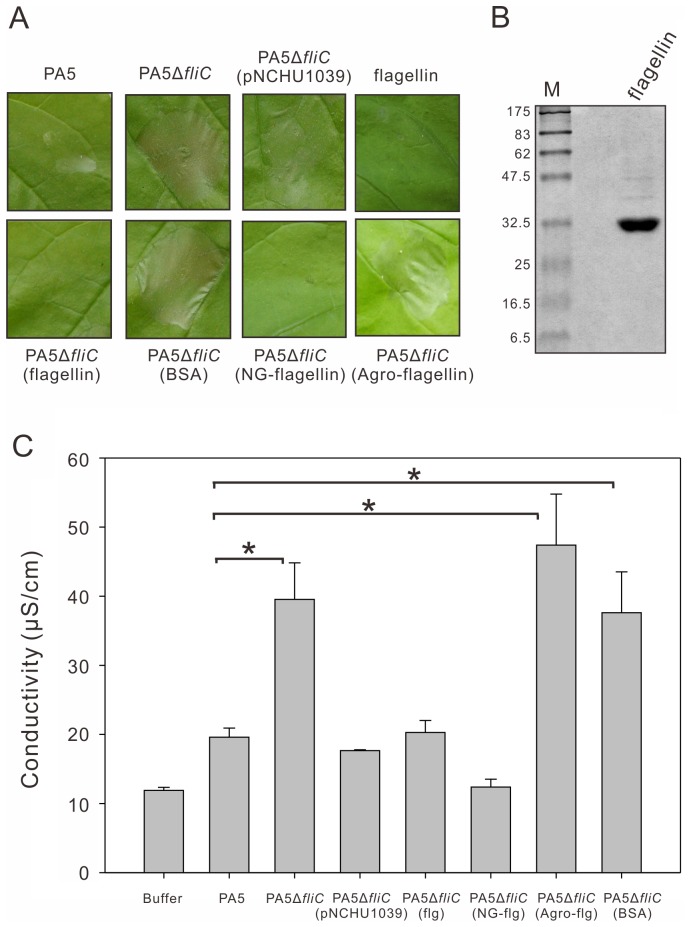
*P. syringae* flagellins or nonglycosylated flagellins can impair the HR elicitation by PA5Δ*fliC*. (A) The macroscopic HR elicition on *Nicotiana benthamiana* leaves. *fliC* mutant (PA5Δ*fliC*) at 1×10^7^ cfu/ml was infiltrated simultaneously with 0.8 µM of *P. syringae* pv. *averrhoi* flagellin, nonglycosylated flagellin (NG-flagellin), *A*. *tumefaciens* flagellin (Agro-flagellin) and BSA proteins, respectively, compared to inoculation with *fliC* mutant (PA5Δ*fliC*) and complemented strain [PA5Δ*fliC* (pNCHU1039)]. The leaf inoculated at concentration of 3.2 µM flagellin by syringe-infiltration was served as a control. The photos were taken at 2 days post inoculation. pNCHU1039: pBBR1MCS5 carrying *fliC*. (B) The *P. syringae* pv. *averrhoi* flagellin protein was purified from PA5 by using ultracentrifugation as described in Materials and Methods and separated by SDS-12% PAGE. The molecular sizes of marker proteins are indicated on the left. (C) Electrolyte leakage from leaf areas inoculated with indicated inoculum. The conductivity values represent the mean and standard error of three different leaves. Significant differences (*p*<0.05) were indicated by asterisk (*). All experiments were repeated at least three times with similar results.

To further investigate that the stronger HR elicited by the *fliC* mutant is due to the lack of flagellin, we performed the HR assay with a complementation of the mutant by transforming a plasmid-borne *fliC*. The complemented strain PA5Δ*fliC* (pNCHU1039) was inoculated into *N*. *benthamiana* at concentration of 1×10^7^ cfu/ml and the HR development was observed at 2 days after inoculation. It elicited weaker HR than PA5Δ*fliC* did, but similar to its wild type did (Figure 5A, upper panel).

To address if there is any effect of flagellin on elicitation of the HR, the purified flagellin of PA5 was simultaneously infiltrated into leaves with PA5Δ*fliC*. PA5 flagellin is ca. 32 kDa in molecular weight, the same size with Pta 6605 flagellin (Figure 5B) [19], and it caused symptom-less in tobacco and *N. benthamiana* when inoculated at a concentration of 3.2 µM flagellin by syringe-infiltration (Figure 5A, far-right upper panel). Compared to the stronger HR on *N*. *benthamiana* elicited by PA5Δ*fliC* or PA5Δ*fliC* mixed with 0.8 µM bovine serum albumin (BSA), PA5Δ*fliC* mixed with 0.8 µM flagellin did not cause necrosis (Figure 5A, lower panel). By contrast, the flagellin of *Agrobacterium tumefaciens* (Agro) which is tolerant by PTI [14], [16] was unable to suppress the HR elicitation by PA5Δ*fliC* (Figure 5A, lower panel). To quantify the intensity of HR, we also measured electrolyte leakage of inoculated leaves at 24 h after inoculation. The PA5Δ*fliC* and PA5Δ*fliC* mixed with 0.8 µM BSA or Agro flagellin induced more leakage of electrolytes, however the complemented strain, PA5Δ*fliC* (pNCHU1039) and PA5Δ*fliC* with 0.8 µM flagellin did not (Figure 5C), suggesting that the flagellin of Pav suppresses the HR on its nonhost plants.

### Glycosylation of PA5 Flagellin is not Necessary for Suppression of the HR and for Virulence at the Post-penetration Stage

**Figure 6:**
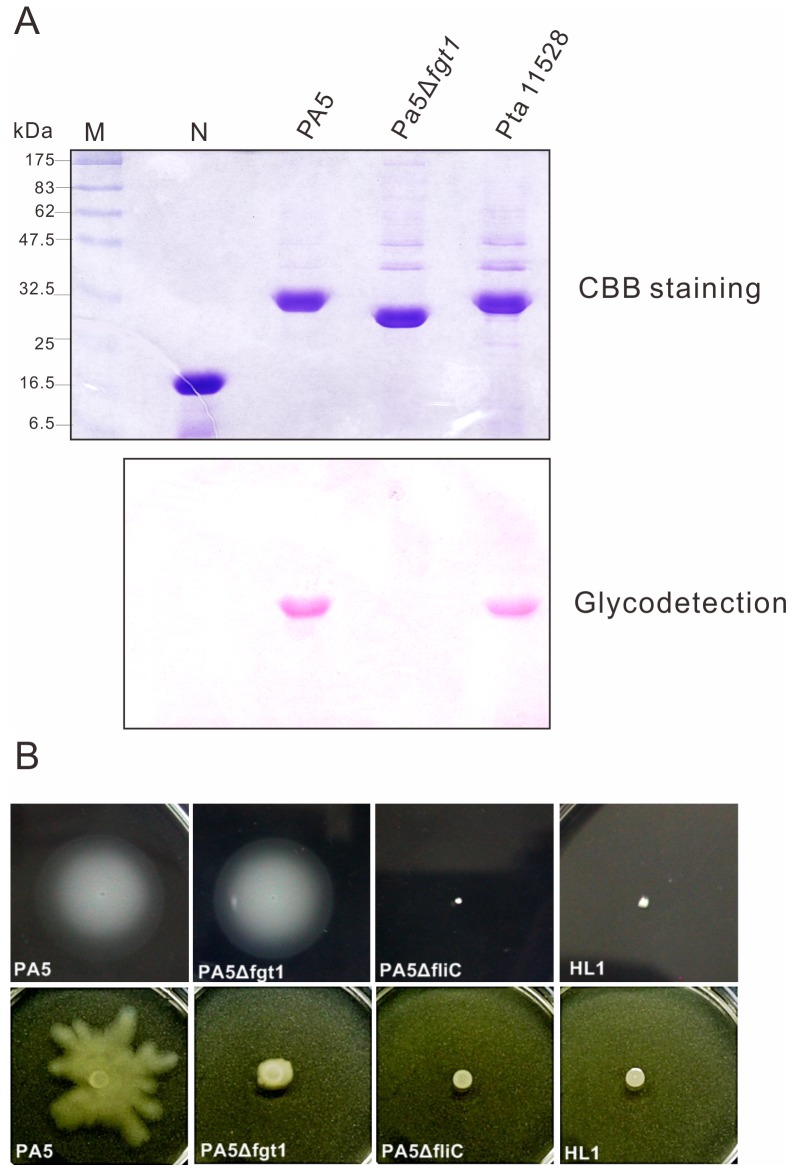
Flagellin glycosylation and motility of wild type (PA5) and the *fgt1* mutant (PA5Δ*fgt1*). (A) Staining on glycosylated flagellin. Purified proteins separated with SDS-12%-PAGE were stained by Coomassie brilliant blue (CBB) (upper panel) and by using a GelCode glycoprotein staining kit (Pierce, Rockford, III) (lower panel). Soybean trypsin inhibitor was served as a negative control (N) and purified flagellin from *P. s.* pv. *tabaci* (Pta) was a positive control. (B) Motility assay. For swimming assays (upper), bacterial strains were incubated for 2 days at 23°C on 0.3% soft agar of *hrp*MM plate. For swarming assays (bottom), bacterial strains were inoculated on 0.5% SWM agar plate and were observed after 24 h at 28°C.

Previous studies showed that glycosylation of flagellin plays a very important role in pathogenesis of Pta [20], [21]. To determine whether glycosylation of Pav flagellin is required for the HR suppression and virulence, we cloned the glycosylation island and deleted one of them, *fgt1*. Using the same strategy as making *fliC* mutants, the *fgt1* gene was deleted by the homologous recombination as depicted in Figure 1A. The PA5Δ*fgt1* was confirmed by PCR using primers prLTail/prOr1–2 and ca. 0.4 kb fragment was amplified as shown in Figure 1C. Flagellin was extracted from PA5Δ*fgt1* by ultracentrifugation and was separated by 12% SDS-PAGE, followed by the detection of glycosylation as described in Materials and Methods. As shown in Figure 6A, the molecular weight of PA5Δ*fgt1* flagellin was decreased about 2–3 kDa compared to its wild type. Also the flagellin proteins purified from PA5 and Pta 11528 (as positive controls) were stained magenta by the reaction kit, but that from PA5Δ*fgt1* was not stained. The results indicate that Pav *fgt1* gene is required for flagellin glycosylation, consistent with the results reported in Pta 6605 and Pgl race 4 [20], [21]. Moreover, to access the effect of flagellin glycosylation on motility, the swimming and swarming assay were performed. Agreed with the results of Pta [20], the PA5Δ*fgt1* showed comparable swimming motility as wild type (Figure 6B, upper panel), whereas it moved slower than wild type did on 0.5% semi-soft agar in the swarming assay (Figure 6B, lower panel).

Inoculation of the non-host tobacco leaves with PA5Δ*fgt1* at 10^8^ cfu/ml resulted in a similar activity of HR to its wild type that elicited a weaker HR at 2.5×10^7^ cfu/ml than PA5Δ*fliC* did (data not shown). Furthermore, the non-glycosylated flagellin purified from PA5Δ*fgt1* was infiltrated into tobacco and *N*. *benthamiana* leaves with 2.5×10^7^ cfu/ml of PA5Δ*fliC* simultaneously. The results of HR necrosis and conductivity assay showed that it still impaired the HR (Figure 5A and C, NG-flagellin), indicating that the glycosylation of PA5 flagellin is not necessary for the suppression of HR. To further investigate the effect of glycosylation on virulence, PA5Δ*fgt1* was inoculated into host carambola leaves by using syringe-infiltration and spray-inoculation. The bacterial multiplication of PA5Δ*fgt1* and PA5Δ*fliC* in host leaves is comparable to wild type (Figure 4B), indicating flagella and flagellin glycosylation are not important for virulence at post-penetration stages. However, the wild type PA5 and its mutants PA5Δ*fliC* and PA5Δ*fgt1* were spray-inoculated on its host leaves, the disease severity caused by PA5Δ*fliC* and PA5Δ*fgt1* was reduced approximately 50% and 30% respectively, compared to the wild type did (Figure S1). The result suggests that motility contributes to accessing infection sites.

### Flagellin Infiltrated into Tobacco Leaves either Simultaneously with Aflagellar HL1 or Prior to the Inoculation with HL1, Both Treatments Impair the HR

**Figure 7:**
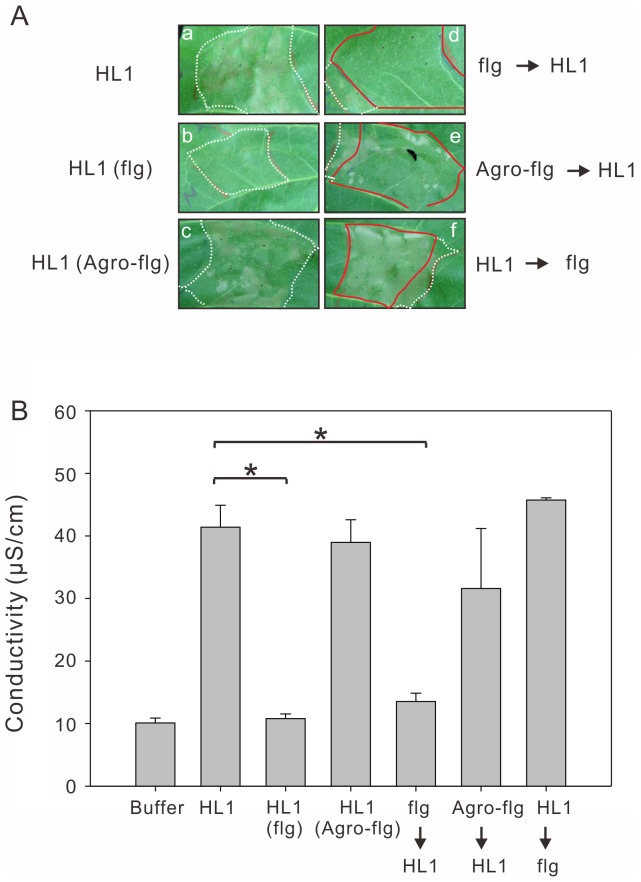
Pre-treatment of flagellin from PA5, but not *A. tumefaciens* (Agro) can impair the HR. (A) 0.8 µM of flagellin from PA5 or Agro was infiltrated simultaneously [indicated as HL1 (flg) and HL1 (Agro-flg), respectively] with 5×10^6^ cfu/ml of HL1 (a, b, c). 6 h prior inoculation with 0.8 µM of flagellins, the leaves were then challenged the HR elicitation by 5×10^6^ cfu/ml of HL1 (d, e). 6 h prior inoculation with 5×10^6^ cfu/ml of HL1, the leaf was infiltrated with 0.8 µM of flagellin (f). The white dotted lines indicate the area infiltrated with HL1 and the red lines indicate the overlapped area infiltrated with HL1 and flagellins. The photos were taken 48 h after inoculation. (B) Electrolyte leakage from leaf areas inoculated with indicated inoculums. The conductivity values represent the mean and standard error of three different leaves. Significant differences (*p*<0.05) were indicated by asterisk (*). All experiments were repeated at least three times with similar results.

To further investigate that the negative effect of flagellin on the HR also exists in the flagellum-deficient strain HL1, we inoculated 5×10^6^ cfu/ml of HL1 containing 0.8 µM flagellin into tobacco leaves and scored visually (Figure 7A) and by electrolyte leakage (Figure 7B). As the effect in PA5Δ*fliC*, the flagellin of PA5 also impaired the HR induced by HL1, whereas the flagellin of *A. tumefaciens* (Agro-flg) had no effect (Figure 7A). Based on the previous research [30], infiltration of a non-pathogen results in basal resistance, which is evident by the failure of a challenge inoculation six hours later to elicit the HR in the tobacco and moreover, the PAMP elicitor flg22 was also able to defeat the challenge of HR elicitation by Pto DC3000 in *N*. *benthamiana* [11]. We infiltrated 0.8 µM flagellin 6 h prior to the inoculation of HL1, and compared to that the same concentration of flagellin was infiltrated 6 h posterior to HL1. As expected, the pre-inoculated flagellin of PA5, but not Agro-flg or flagellin infiltration 6 h later, impaired the HR induced by HL1 (Figure 7). Together with results showing that the flagellin of *A. tumefaciens* was unable to induce PTI in tobacco [11], [14] and didn’t suppress the HR elicitation (Figure 5A), it indicates that suppression of HR is due to the PTI induced by flagellin proteins of plant pathogens. In addition, we also determined the ability of HR eliciting by a wild type PavHL1 and a *fliC*-overexpressing strain HL1 (pNCHU1039) on tobacco leaves. The tested bacteria were induced on *hrp*MM for 6 h before infiltrated into tobacco leaves. Although the *fliC* was expressed in *hrp*MM, the motility was not recovered (Figure 2B) and flagellins in HL1 (pNCHU1039) were not detected in the supernatant fraction (data not shown). Both strains showed a comparable HR symptom at 12 hpi (Figure S2), indicating that overexpressed FliC in HL1 (pNCHU1039) has no effect on HR elicitation as flagellin is not secreted.

### The HR Elicited by PA5 and PA5Δ*fliC* was Boosted by Inhibition of Innate Immunity with Cycloheximide Treatment

**Figure 8:**
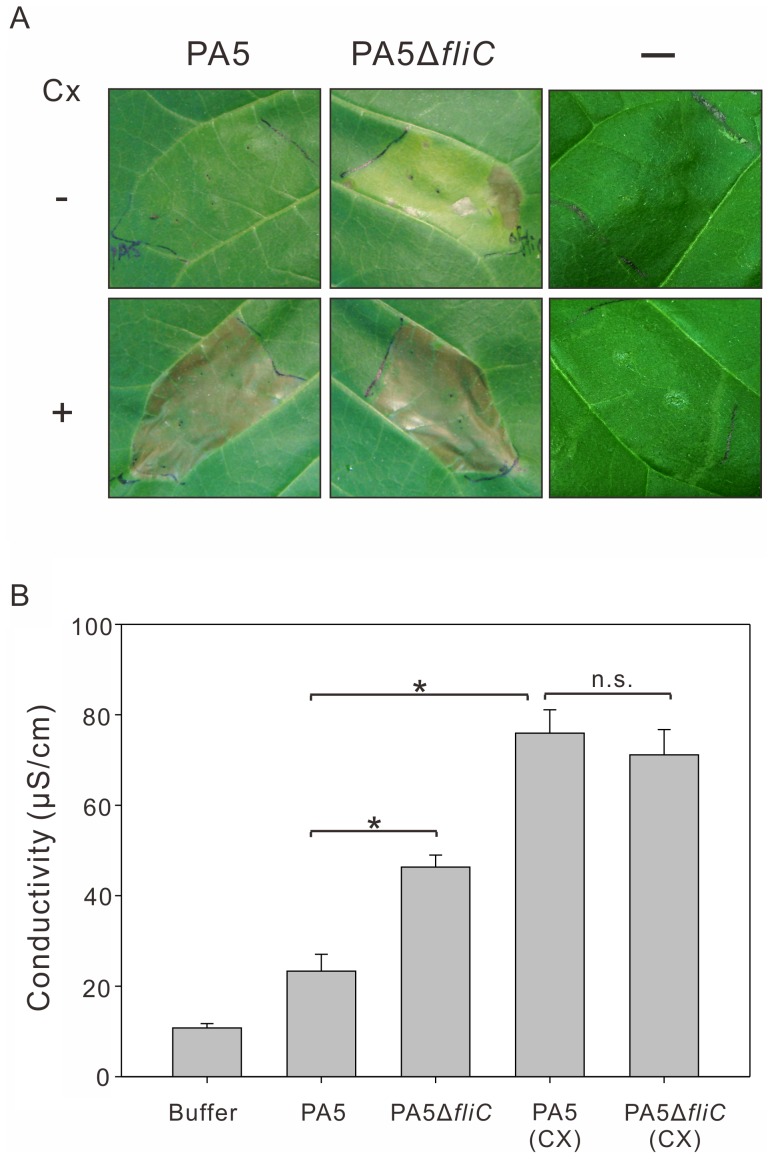
Inhibition of innate immunity with cycloheximide treatment. (A) PA5 and PA5Δ*fliC* were infiltrated at 2×10^7^ cfu/ml [or with 5 µg/ml cycloheximide (Cx)] in tobacco leaf and photographed at 2 days after inoculation. Inoculation buffer (-) or 5 µg/ml cycloheximide infiltrated alone was served as a control. (B) Electrolyte leakage from leaf areas inoculated with indicated inoculum. Significant differences (*p*<0.05) were indicated by asterisk (*). n.s., not significant differences (*p*>0.05).The experiment was repeated at least three times with similar results.

A previous research indicated that the development of plant basal resistance could be suppressed by 5 µg/ml cycloheximide which at that concentration could not affect the development of the HR [30]. In this work, we infiltrated 2.5×10^7^ cfu/ml of bacteria, PA5 and PA5Δ*fliC* along with 5 µg/ml cycloheximide into tobacco leaves. Inoculation buffer (Figure 8A. far-right on upper panel) and only 5 µg/ml cycloheximide treatment (Figure 8A. far-right on bottom panel) were served as a control. As shown in Figure 8A, the HR symptoms appeared quickly at 12 hpi and were stronger when mixed with cycloheximide in the inoculums, especially in the inoculation of PA5 which usually induces a delayed HR. Those results were also quantified and confirmed by measuring electrolyte leakage in conductivity assays (Figure 8B). Once again, it is evident that the Pav flagellin is one of the PAMPs contributed to inducing PTI which can suppress the HR induced by incompatible pathogens.

## Discussion

A collection of *P. syringae* pv. *averrhoi* (Pav) strains isolated from different area and host varieties in Taiwan were surveyed for their swimming ability and HR elicitation on nonhost plants in this study. Interestingly, the strains which are motile often elicit the weaker HR on their nonhost plants. For instance, the tested strains PA5, HL2 and HL9 are motile but elicit the delayed HR, in contrast to aflagella HL1 strain which elicits a very strong HR. Moreover, flagellin and its glycosylation of *P. syringae* pv. *tabaci* (Pta) and *glycinea* have been reported to be involved in the induction of plant defense system or in the determination of host specificity [20], [21], [31]. Therefore, it leads us to explore the role of Pav flagellin played in incompatible interactions between Pav and its nonhost plants. By cloning and sequence analysis of Pav *fliC* gene and its related glycosylation island, our results reveal that the amino acid sequence of FliC from Pav PA5 is identical to *P. s.* pvs. *tabaci*, *glycinea* and *phaseolicola*, and that the gene organization and sequence of glycosylation island are highly conserved in *P. syrinage* pathovars. According to mutagenesis of PA5 *fliC* and glycosyltransferase-coding *fgt1* located in the glycosylation island, data indicate that *fliC* in Pav is a single gene encoding flagellin and is indispensable for bacterial motility and *fgt1* is responsible for glycosylation of flagellin and swarming but not for swimming ability suggesting that the flagellin glycosylation is important for Pav moving on solid surface. Furthermore, based on the results of inoculations on nonhost leaves with PA5 vs PA5Δ*fliC*, flagellin mixed with PA5Δ*fliC* or with HL1, and flagellin prior to the inoculation of HL1, it is evident that Pav FliC suppresses the HR elicitation, and this suppression does not require glycosylation of flagellin. In another inoculation test by adding 5 µg/ml cycloheximide (a plant protein synthesis inhibitor and proved to be an inhibitor of plant basal resistance [30]) to an inoculum of PA5, the remarkable symptom of HR on tobacco leaves appeared, suggesting that HR suppression is due to a basal resistance (BR) induced by flagellin, that phenomenon is consistent with statements by Klement *et al*. [10], [30] and Collmer and his coworker [11]. That is, the BR (also currently known as PTI) triggered by PAMPs such as LPS and flg22, heat-killed pathogens, saprophytic bacteria, and *hrp* mutants of *P. syringae* is able to prevent HR when the induction time of HR is longer or later than the time required for the development of BR [10], [11], [30]. In comparing to Pta, however, we also found distinct biological features of Pav flagellin. Compared to enhanced growth of PtaΔ*fliC* in nonhost plants (including tomato and Arabidopsis) and reduction of virulence on its host tobacco [31], [32], the growth of PavΔ*fliC* in nonhost tobacco leaves is not promoted and this mutant grows as well as its wild type does on host plants. Further, PA5Δ*fgt1* which possesses nonglycosylated flagella behaves like its wild type in the interactions with host at post penetration stage and with nonhost plants, in contrast to reduced virulence of *P. syringae* pv. *glycinea fgt1* mutant on its host plant soybean [21]. Overall, flagellin and its glycosylation in Pav are not involved in host specificity.

A conserved N-terminal peptide of flagellin, flg22, can function as an elicitor to induce PTI in tomato and *Arabidopsis* [14], [33]. This defense response also known as BR (or early induced response, EIR) induced by LPS, flg22, or *hrp* mutants results in suppression of HR on tobacco and *N*. *benthamiana* when challenge inoculated with incompatible *P. syringae* pathovars. This reveals that when BR expresses early, HR elicitation by incompatible pathogens is inhibited and the inoculated area remains symptomless [10], [11]. In this study, we also demonstrated that the flagellin is able to induce BR within 6 hr at 25–28°C according to that the HR elicited by aflagella HL1 was suppressed prior to the inoculation with flagellins (Figure 7). Moreover, based on the results of several inoculation assays with flagella vs aflagella bacteria and cycloheximide treatments, the flagella of Pav PA5 are able to quickly induce BR in non-host plant, resulting in the suppression of HR development. Thus the HR elicited by PA5 is reduced or delayed. Hence, the biological features of Pav demonstrated here may explain that the intensity of HR elicitation (e.g by HL1 vs PA5) occurs among Pav strains isolated from the field. In addition, when PA5Δ*fliC* mixed with purified flagellin of *A. tumefaciens* was infiltrated into nonhost leaves, the HR is not repressed. It further confirms that neither the flg15^A.tum^ nor intact flagellin of *A. tumefaciens* is able to induce plant immunity in the tobacco [11], [14]. The non-glycosylated flagellins isolated from Pav PA5Δ*fgt1* are still able to suppress the HR, thereby the conserved domain of N-terminus flagellin [34], but not the glycosylated domain which was identified in the flagellin of Pta 6650 [20], is a decisive target for the recognition by plant receptors, for example FLS2 in *Arabidopsis*.

In a single inoculation of incompatible pathogens on tobacco, two plant responses, BR and HR, develop in a parallel manner. They may interact with each other in the few hours in pathogenesis [30]. However, when the BR develops before the induction of the HR, the inoculated plants remain symptomless [10], [30]. In other word, the length of the induction period may be critical in determining whether or not HR occurs quicker in certain circumstances [35]. The mechanism of action of BR to prevent HR is not clear yet. Several possibilities might be debated. First, the bacteria were inactivated by BR so that a few bacteria translocate TTSS effectors to elicit the macroscopic HR. A model was proposed that one bacterium is sufficient to trigger the death of a plant cell resulting microscopic HR. However, to cause macroscopic HR needs at least four bacteria to one plant cell [36]. Second, the basal defense system may influence the HR-based defense due to the inhibition of *hrp* genes expression and bacterial metabolism during BR development [30], [35]. Third, the expressing profiles between BR and HR in plants are known to be overlapped [37], thus the competition between these pathways probably occurs in the pathogen infection. Uncovering those puzzles will be worthy to be investigated with using PA5 and its *fliC* mutant as isogenic strains.

One role of TTSS effectors in pathogenesis is the suppression of PTI and ETI. A growing number of effectors are known to suppress HR-associated cell death and to suppress PTI [38]. We have cloned and sequenced the pathogenicity island from Pav HL1. Interestingly, data reveal that some open reading frames located in those loci are interrupted (for example: *hrpW1*, *hopM1*, and *hopAA1-1*) or inserted by a transposon (*hopA2*) [39]. In Pto DC3000, HopM1 was shown to disrupt plant immunity by targeting an immunity-associated protein, AtMIN7, in *Arabidopsis thaliana* and results in its degradation via host proteasome [40]. HopAA1-1 was also shown to suppress the flg22-dependent *NHO1* induction in *Arabidopsis* [16]. HopA2 is homologous to HopA1 which was also indicated involving in altering basal defense by inducing JA-responsive genes [41] and can suppress PTI-associated ROS production [42]. The HrpW1 belongs to the harpin family which probably facilitates the TTSS pilus to pass through plant cell wall and overcomes the plant basal defense [43], [44]. These effector proteins from Pto were all demonstrated involving in PTI suppression but they are not functional in Pav. Whether those nonfunctional effectors cause Pav not to efficiently defeat the PTI observed here remains unknown.

The involvement of Pav flagellum in virulence described in this study is consistent to the findings that the importance of flagellar motility in pathogenicity of *P. syringae* pathovars is prior to and during infection on host plants whereas flagella are generally not required for virulence at post-penetration stages [16], [24], [25]. One exception for that role was reported for Pta, in which flagellum mutants showed reduced virulence, even after direct infiltration into tobacco leaves [17]. Moreover, Ichinose and his coworkers first described that the glycosylation of flagella in Pta and Pgl determines the host specificity and also affects the virulence prior to infection on host plants [20], [21]. In this study, the glycosylation of Pav flagellin is not involved in the suppression activity on the HR, but does reduce the virulence prior to pathogen penetration, as Pta does [20]. The reduced infectivity could be resulted from that the flagellin glycans influence the flagellar swarming and adhesion on the surface of host tissues [20].

From our observation presented here, at least two strains isolated from the field are non-motile on NGA soft agar plate and one of them, HL1, was demonstrated that it’s flagellum-defective. Is it a bias for Pav to evolve to be flagellum-deficient that does not affect its survival in the field? A recent research revealed the flg22 caused dramatic stomatal closure in *Arabidopsis* plants, suggesting that PAMP-induced stomatal closure is a common defense in many plants [45]. Here, we demonstrated that Pav flagellin is a major PAMP molecule to activate plant innate immunity which is able to suppress HR. Therefore, it is hinting that Pav probably exploits defective flagellum to escape the defense of stomatal cells and the first line of BR-based defense, and to get into the plant successfully. Also, to realize how many and which effectors of Pav involved in the PTI suppression is necessary in the future.

## Materials and Methods

### Bacterial Strains, Plasmids, and Culture Conditions

Bacterial strains and plasmids used in this study are listed in Table 2. *Escherichia coli* was grown in Luria-Bertani (LB) [46] at 37°C and *P. syringae* was grown in King’s B (KB) [47] at 30°C. For the swimming assay, bacteria were picked from a KB agar plate with a sterile toothpick and spotted onto 0.3% soft agar plates, including nutrient gelatin agar (NGA) [48], LB medium containing 10 mM MgCl_2_ (LB-MgCl_2_), or *hrp* minimal medium (*hrp*MM; 10 mM fructose, 10 mM mannitol, 50 mM potassium phosphate buffer, 7.6 mM (NH_4_)_2_SO_4_, 1.7 mM MgCl_2_ and 1.7 mM NaCl, pH 5.7) [49], then incubated at 25°C for 2 days. Antibiotics were used at the following concentrations (micrograms per milliliter): ampicillin, 100; cycloheximide, 2; gentamycin, 10; kanamycin (Km), 50; and nalidixic acid, 20.

**Table 2:** Bacterial strains and plasmids used in this study.

Designation	Relevant characteristics	Source or Reference
**Strains**		
*E. coli*		
DH10B	*endA1 hsdR17 recA1 relAD* (*argF-lacZYA*) U169 f80d *lacZ*DM15	Life Sciences Technologies
S17-1	*thi pro hsdr^-^ hsdM^+^ recA* [*chr*::PR4-2-Tc::Mu- Km::Tn7]	[56]
*P. s.* pv.averrhoi		
HL1	Wild type isolated from carambola, fruit, Juolan.	[23]
HL2	Wild type isolated from carambola, leaf, Juolan.	[23]
HL9	Wild type isolated from carambola, leaf, Juolan.	[23]
PA5	Wild type isolated from carambola cv. Malaysia, fruit, Guoshing.	[23]
HL2Δ*fliC*	*fliC* mutant generated by pMC	This study
HL9Δ*fliC*	*fliC* mutant generated by pMC	This study
PA5Δ*fliC*	*fliC* mutant generated by pMC	This study
PA5Δ*fgt1*	Glycosylation island *fgt1* mutant	This study
*P. s.* pv. tomato		
DC3000	Wild type, Rif^r^	[57]
CUCPB5467	*fliC* mutant	Kvitko, B.H. and Wei, C.-F.
*P. s.* pv. tabaci 11528	Wild type	American Type Culture Collection
*Agrobacterium tumefaciens* LBA4404	Derived from the wild-type Ach5, containing a virulence plasmid 5	Clontech Laboratories
**Plasmids**		
pBBR1MCS5	A broad host range vector containing *lac* promoter, compatible to IncP, IncQ, or IncW group plasmids, Gm^r^	[58]
pCR-XL-TOPO	Cloning vector for PCR product, Amp^r^, Km^r^	Invitrogen
pK18*mobsacB*	Small mobilizable vector, Km^r^, sucrose-sensitive (*sacB*)	[59]
pMC	1.87 kb chimeric PCR product deleting *fliC* cloned into pK18*mobsacB* at *Eco*RI site, Km^r^	[32]
pNCHU1039	0.84 kb *P. syringae* pv. averrhoi *fliC* cloned in pBBR1MCS5	This study
pNCHU1067	2.0 kb chimeric PCR product deleting *fgt1* cloned into pK18*mobsacB* at *Bam*HI-*Hin*dIII site	This study

### Recombinant DNA Techniques

DNA manipulations were done according to standard procedures [50]. Plasmids were introduced into bacteria by electroporation (GenePulser, Bio-Rad, Richmond, Calif.) or triparental mating [51]. PCRs using KlenTaq (Protech) or *Pfu* (Invitrogen) enzymes were performed according to standard procedures [50]. Long-PCR was carried out with Elongase (Invitrogen). Primers used in this study are listed in Table S1. Database searches were performed using gapped BLASTN and BLASTP (htp//www.ncbi.nlm.nih.gov/).

### Construction of *fliC* Mutant and *fgt1* Mutant

To generate *fliC* mutant, a pK18*mobsacB* derivative plasmid pMC [32] was applied to create Pav *fliC* mutant as depicted in Figure 1A. It was transferred from *E*. *coli* S17-1 to *P. syringae* pv. *averrhoi* (Pav) by conjugation and independent transconjugants were selected. Bacterial cells were plated onto a KB plate containing 10% sucrose and incubated for 48 h at 30°C. The growing colonies were selected for Km-sensitivity, indicating the excision of plasmid by a second crossing-over event. Subsequently, *fliC* deletion was confirmed by PCR using a primer pair PC1/PD2, that 1.5 kb fragment could be amplified from *fliC* mutant (Figure 1B). To make the *fgt1* mutant, PCR and homologous recombination were carried out as shown in Figure 1A. Approximately 1-kb fragments flanking with *fgt1* gene was amplified by PCR with the primers prDOR1-1/prDOR1-2 and prDOR2-1/prDOR2-2, respectively and cloned into pK18*mobsacB*. The resulting plasmid pNCHU1067 was transformed into *E*. *coli* S17-1 and as following by the same method as described above to delete the *fliC* gene in Pav PA5. The *fgt1* mutant was isolated and confirmed by PCR using a primer pair, prLTail/prOR2-1 (Figure 1C).

### Electron Microscopy Observation

Bacteria were incubated on KB plates at 30°C for 2 days and then resuspended with ddH_2_O to OD_600_ of 0.3 and placed on grids. Grids were then subjected to a routine negative staining in 2% uranyl acetate (Sigma) for 30 s and observed with a transmission electron microscope (JEM-1200CX II, JEOL, Tokyo, Japan).

### Motility Assay

For swimming assay, bacteria were picked from a KB agar plate with a sterile tooth pick and spotted onto 0.3% soft agar of *hrp*MM or LB containing 10 mM MgCl_2_ plates and were incubated for 2 days at 23°C. Swarming assays were performed on SWM plate (0.5% peptone, 0.3% yeast extract and 0.5% Eiken agar; 20). 2 µl of bacterial strains at 1 OD_600_ were inoculated on 0.5% SWM agar plate and were observed after 24 h at 28°C.

### Plant Materials, Bacterial Inoculations and Virulence Assays

Fully expanded and healthy leaves of 8-week post-germination tobacco (*Nicotiana tabacum* L. cv. Van-Hicks) and 6-week post-germination *N. benthamiana*, 4-week post-germination tomato (*Solanum lycopersicon* cv. Moneymaker) were used for HR assays. The plants were grown under greenhouse conditions at 23–25°C with 16 h illumination, and then transferred to the laboratory 1 day prior to inoculation with a blunt syringe. Starfruit (*Averrhoa carambola*) plants were grown under greenhouse condition at 28°C. For HR assays, bacteria were grown overnight on KB agar supplemented with appropriate antibiotics and resuspended in 10 mM MgCl_2_ at a cell density of 1×10^8^ cfu/ml, then diluted to indicated concentrations. Inoculations were performed by pricking leaves with a dissecting needle and then pressing the blunt end of the tuberculin syringe against the leaf surface while supporting the leaf with a finger [52]. The development of HR was observed within 24 h at room temperature. To measure bacterial growth in planta, three leaf disks from tobacco or starfruit leaves were ground in 300 µl of 10 mM MgCl_2_ and serial dilutions were spotted onto KB medium containing nalidixic acid and cycloheximide. Bacterial colony forming units (CFU) were counted 2 days after incubation at 30°C. Each data point is measured with triplicates. For infectivity assay on host starfruit leaves using a spray-inoculation, the tested bacteria were suspended in distilled water containing 0.025% Silwet L77 to a density of 0.3 OD_600_. The leaves inoculated on both leaf surfaces were covered with plastic bags for 6 days and the symptom severity was scored 15 days post inoculation. Symptom formation was scored with 0–10 scales according to the percentage of diseased area appearing on the leaf: The scales are: 0, 0%; 1, 1–3%; 2, 3–6%; 3, 6–12%; 4, 12–25%; 5, 25–50%; 6, 50–75%; 7, 75–88%; 8, 88–94%; 9, 94–97%; 10, 97–100%.

### Flagellin Purification and Glycoprotein Detection

*P. syringae* pathovars were grown in LB with 10 mM MgCl_2_ for 48 h at 25°C. Collected cells were transferred to *hrp*MM and further incubated for 24 h at 23°C. After centrifugation at 7,000 *g* for 10 min, the bacterial pellet was resuspended in phosphate buffer (50 mM sodium phosphate, pH 7.0). Bacterial flagella were sheared from cells by vortexing for 1 min and collected by centrifugation at 10,000 *g* for 30 min. The resultant supernatant was further centrifuged at 100,000 *g* for 30 min [53]. The flagellar pellet was suspended in ddH_2_O and adjusted to 0.8 µM for inoculation.

For detection of glycoproteins, the purified flagellin proteins from Pav PA5 or PA5Δ*fliC* were separated by SDS-PAGE and subjected to the GelCode glycoprotein staining kit (Pierce, Rockford, IL, U.S.A.) according to the manufacturer’s instruction.

### Overexpression of Pav *fliC* Gene in *E. Coli* and Preparation of Anti-FliC Antibody

The FliC protein was overexpressed by the T7 polymerase dependent expression method [50]. In brief, *E. coli* BL21 (DE3) harboring pNCHU1001 (pET29a::*fliC*), was grown in LB broth to an OD_600_ of 0.5–0.6 at 37°C, induced with 0.5 mM isopropyl β-D-thiogalactopyranoside (IPTG) for 2 h. Bacterial cells were collected by centrifugation, resuspended in 2× loading buffer, sonicated (Heat system Ultrasonics XL-2020 Sonicator) for one min, and heated to 100°C for 5 min before being subjected to electrophoresis. The FliC protein was purified with an Elutrap apparatus as described in previous study [54] and the purified proteins were used to raise polyclonal antibodies in rabbit following standard procedures [55].

### Immunoblot Analysis

Bacteria were separated into cells and supernatant by centrifugation at 2,000 *g* for 10 min. The cell pellets were washed with extraction buffer [50 mM Tris-HCl pH 8.0, 150 mM NaCl, 0.1% Tween-20 (v/v), 10% glycerol (v/v), 1 mM phenylmethylsulphonyl fluoride (PMSF)], resuspended in 1 ml of the same buffer, sonicated, precipitated with 5% TCA on ice for 1 h, and dissolved in 100 µl of extraction buffer. Total proteins were separated by 12% SDS-PAGE and were electrotransferred to Immobilon-P membranes (PVDF, Millipore) in a semi-dry transfer unit TE-70 (Hoefer) following the manufacturer’s instructions. The membranes were probed with the preabsorbed anti-FliC antibody at a ratio of 1 to 500 followed by alkaline phosphatase-conjugated anti-rabbit IgG antibody (Boehringer Mannheim) and stained with CDP-Star® (Applied Biosystems) as described by the manufacturer.

### Electrolyte Leakage Assay

For electrolyte leakage assays, six leaf disks were excised with a 0.6-cm-diameter cork borer from inoculated area in at least three different leaves. Samples were placed in a 30 ml glass test tube containing 10 ml of distilled water. The tubes were shaken at 200 rpm at room temperature for 1 h. Then all leaf disks were removed and the conductivity of the bathing solution was measured using IONcheck30 (Radiometer analytical, France) with a probe CDC30T.

## Supporting Information

Figure S1 **Infectivity assay.** (A) The bacterial spot symptoms caused by PavPA5 and its *fgt1* and *fliC* mutants. The indicated strains were spray-inoculated onto starfruit leaves as described in Materials and Methods. Photos were taken 15 days post inoculation. CK: The leaf was sprayed with water containing 0.025% Silwet L77 as a negative control. (B) The disease index was measured based on the severity of spot symptoms which was scored following the criteria as described in Materials and Methods. Significant difference (*, *p*<0.05 or *p* = 0.07) was indicated.
(TIF)

Figure S2 **The hypersensitive response of tobacco leaves elicited by HL1 and HL1 (pNCHU1039).** Two bacterial strains showed the comparable symptoms on nonhost plant. After grown in KB medium overnight, the indicated strains were cultured in *hrp*MM for 6 hrs and then adjusted the concentration to 10^7^ cfu/ml. Subsequently, they were infiltrated into the tobacco leaf with a blund syringe and photographed at 12 h post inoculation. pNCHU1039: pBBR1MCS5 carrying *fliC* gene.
(TIF)

Table S1 Primers used in this study.
(DOC)

Table S2 Comparison of flagellum-related gene products from *P. s.* pv. *averrhoi* (AC EF544881) with their homologs from other *P. syringae* pathovars.
(DOC)
