# Supplementary figures and images for: Plant Innate Immunity Induced by Flagellin Suppresses the Hypersensitive Response in Non-Host Plants Elicited by Pseudomonas syringae pv. averrhoi
Source: PLoS One. 2012 Jul 23;7(7):e41056. doi: 10.1371/journal.pone.0041056 (PMC3402453; doi:10.1371/journal.pone.0041056)

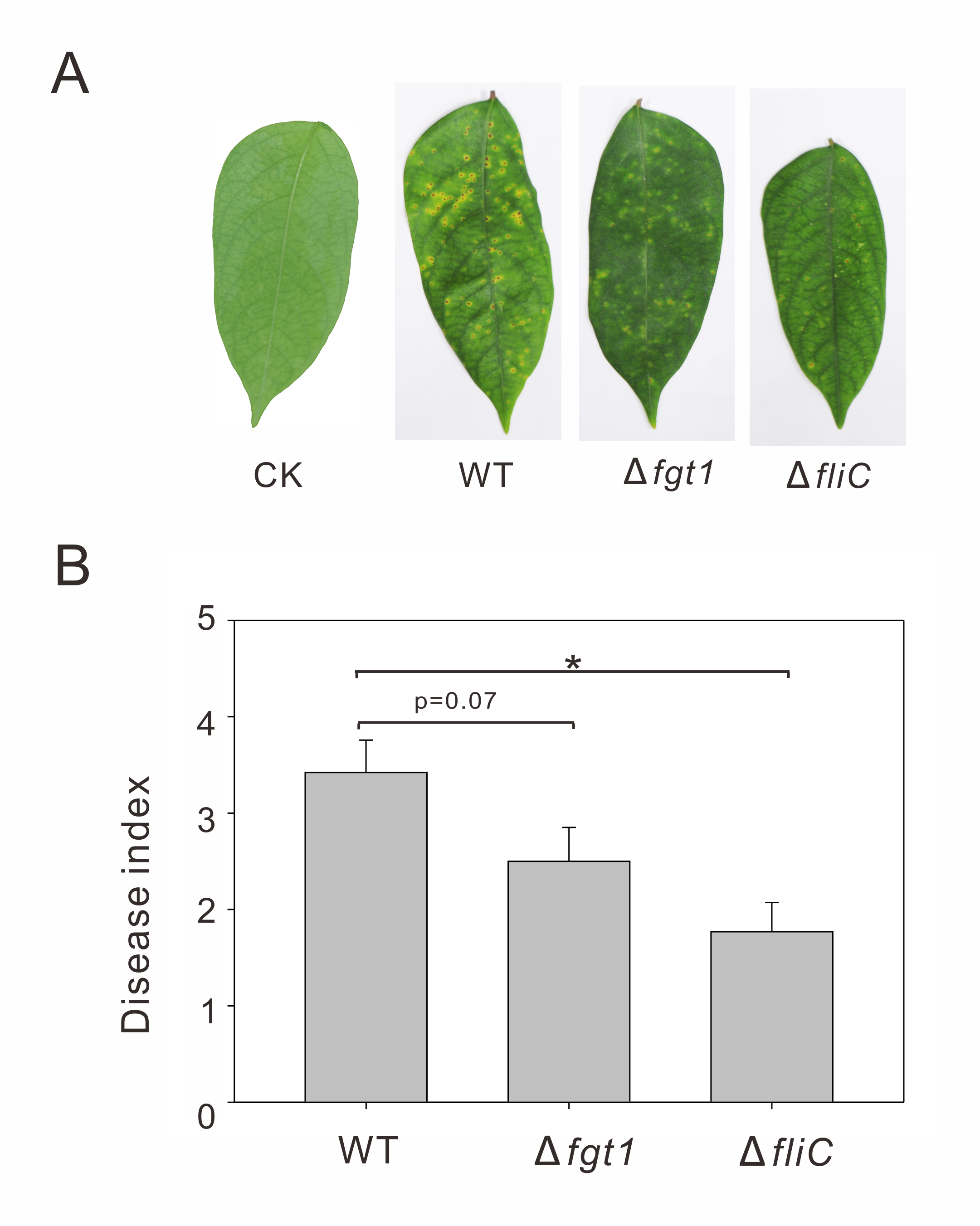

Supplement: Figure S1 — Infectivity assay. (A) The bacterial spot symptoms caused by PavPA5 and its fgt1 and fliC mutants. The indicated strains were spray-inoculated onto starfruit leaves as described in Materials and Methods. Photos were taken 15 days post inoculation. CK: The leaf was sprayed with water containing 0.025% Silwet L77 as a negative control. (B) The disease index was measured based on the severity of spot symptoms which was scored following the criteria as described in Materials and Methods. Significant difference (*, p<0.05 or p = 0.07) was indicated. (TIF) [file pone.0041056.s001.tif]

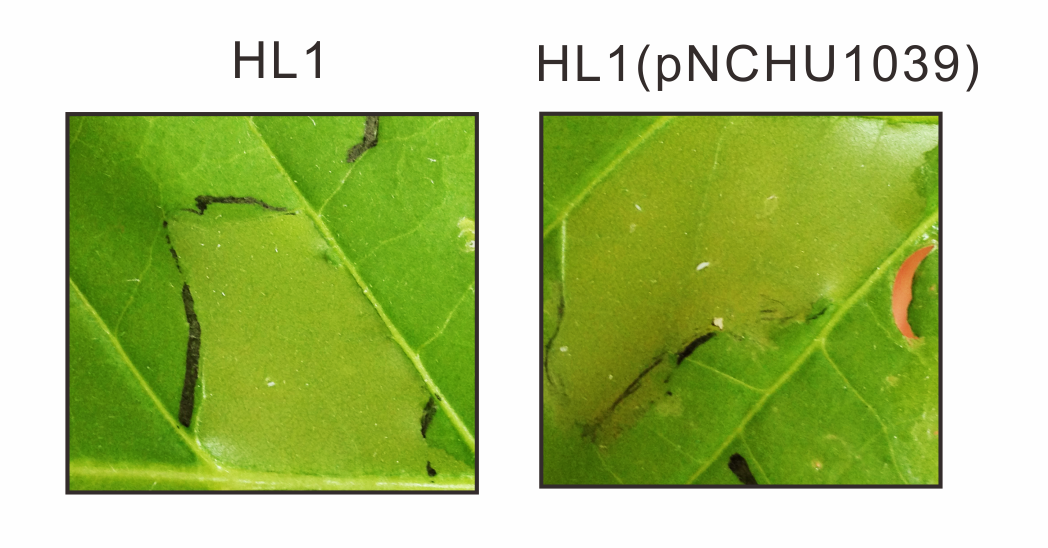

Supplement: Figure S2 — The hypersensitive response of tobacco leaves elicited by HL1 and HL1 (pNCHU1039). Two bacterial strains showed the comparable symptoms on nonhost plant. After grown in KB medium overnight, the indicated strains were cultured in hrpMM for 6 hrs and then adjusted the concentration to 107 cfu/ml. Subsequently, they were infiltrated into the tobacco leaf with a blund syringe and photographed at 12 h post inoculation. pNCHU1039: pBBR1MCS5 carrying fliC gene. (TIF) [file pone.0041056.s002.tif]
