# Supplementary material for: Plant Innate Immunity Induced by Flagellin Suppresses the Hypersensitive Response in Non-Host Plants Elicited by Pseudomonas syringae pv. averrhoi
Source: PLoS One. 2012 Jul 23;7(7):e41056. doi: 10.1371/journal.pone.0041056 (PMC3402453; doi:10.1371/journal.pone.0041056)
Supplement: Table S1 — Primers used in this study. (DOC) [file pone.0041056.s003.doc]

**Supporting information**

**Table S1.** Primers used in this study

| Primer name | Primer sequence | Restriction enzyme |
| --- | --- | --- |
| prfliC-f | 5’-GCGCATATGGCTTTAACAGTAAAC-3’ | *Nde*I |
| prfliC-r | 5’-GACGGATCCTTACTGAAGCAGTTTC-3’ | *Bam*HI |
| prLTail | 5’-GTCCAAGCTGAACCTGTTCAGCCAG-3’ | － |
| prCHead | 5’-GCTACGTTGGTGTTTACTGTTAAAG-3’ | － |
| prCDS-1 | 5’-GTTGGATCCGCGGTACTGAAAC-3’ | － |
| prCDS-2 | 5’-GTTTCAGTACCGCGGATCCAAC-3’ | － |
| prfliS-m-f | 5’-GATCAGAAGATGCTCG-3’ | － |
| prfleS-585-r | 5’-GCGATCCTTGAGCCG-3’ | － |
| prflgJ-437-f | 5’-GTCCGCTGTGGGCC-3’ | － |
| prflgL-m-r | 5’-GAAGGTGAAGGTCTG-3’ | － |
| prDOR1-1 | 5’-TCAGGTACCTGCAATATTGGGCTAAC-3’ | *Bam*HI |
| prDOR1-2 | 5’-ATAGGTACCAGACCTTCACCTTCCG-3’ | *Bam*HI |
| prDOR1-3 | 5’-TGTGGATCCACACAGAAACTTGATG-3’ | *Bam*HI |
| prDOR1-4 | 5’-TAGAAGCTTCCATCTGCACCTG-3’ | *Hin*dIII |
| prPC1 | 5’-TGCGTTCGACATTTCACTGG-3’ | － |
| prPD2 | 5’-CGGGATCCTCTCCCTTCAAGAATGTCGC-3’ | *Bam*HI |
| prOR1-2 | 5’-TATGGATCCAGCCATCAGTGACTG-3’ | *Bam*HI |
