# Supplementary material for: Plant Innate Immunity Induced by Flagellin Suppresses the Hypersensitive Response in Non-Host Plants Elicited by Pseudomonas syringae pv. averrhoi
Source: PLoS One. 2012 Jul 23;7(7):e41056. doi: 10.1371/journal.pone.0041056 (PMC3402453; doi:10.1371/journal.pone.0041056)
Supplement: Table S2 — Comparison of flagellum-related gene products from P. s. pv. averrhoi (AC EF544881) with their homologs from other P. syringae pathovars. (DOC) [file pone.0041056.s004.doc]

**Table S2.** Comparison of flagellum-related gene products from *P. s.* pv. *averrhoi* (AC EF544881) with their homologs from other *P. syringae* pathovars.

| Pathovars^e^ | FlgK  (686) ^a^ | FlgL  (530) | Fgt1  (1191) | Fgt2  (968) | Orf3  (308) | FliC  (282) | FlaG  (130) | FliD  (492) | FliS  (132) |
| --- | --- | --- | --- | --- | --- | --- | --- | --- | --- |
| glycinea race 4 | __ ^d^ | 97 ^b^ /98 ^c^ | 98/99 | 98/98 | 99/99 | 100/100 | 99/99 | 97/98 | 100/100 |
| tabaci 6605 | 99/99 | 99/99 | 98/99 | 97/98 | 99/99 | 100/100 | 99/99 | 97/98 | 100/100 |
| phaseolicola 1448A | 99/99 | 99/99 | 98/99 | 97/98 | 99/99 | 100/100 | 99/99 | 97/98 | 100/100 |
| tomato DC3000 | 96/99 | 94/98 | 92/96 | 90/94 | 98/98 | 95/98 | 93/94 | 94/97 | 97/98 |
| syringae B728a | 85/94 | 82/90 | 82/90 | 76/86 | 97/97 | 90/95 | 85/89 | 80/89  (483) | 93/97 |
| pisi race 1 | __ | __ | __ | __ | __ | 93/96 | __ | __ | __ |
| syringae 61 | __ | __ | __ | __ | __ | 93/97 | __ | __ | __ |

a: Total amino acid of protein.

b: Percentage of amino acids in identity.

c: Percentage of amino acids in similarity.

d: No sequence available.

e: References: *glycinea* race 4 [1]; *tabaci* 6605 [2]; *phaseolicola* 1448A [3]; *tomato* DC3000 [4]; *syringae* B728a [5]; *pisi* race 1 [6]; *syringae* 61 (AC EF544882).

1. Takeuchi K, Taguchi F, Inagaki Y, Toyoda K, Shiraishi T, et al. (2003) Flagellin glycosylation island in *Pseudomonas syringae* pv. *glycinea* and its role in host specificity. J Bacteriol 185: 6658-6665
2. Taguchi F, Takeuchi K, Katoh E, Murata K, Suzuki T, et al. (2006) Identification of glycosylation genes and glycosylated amino acids of flagellin in *Pseudomonas syringae* pv. *tabaci*. Cell Microbiol 8: 923-938.
3. Joardar V, Lindeberg M, Jackson RW, Selengut J, Dodson R, et al. (2005) Whole genome sequence analysis of *Pseudomonas syringae* pv. *phaseolicola* 1448A reveals sequence divergence among pathovars in genes involved in virulence and mobile genetic elements. J Bacteriol 187: 6488-6498.
4. Buell CR, Joardar V, Lindeberg M, Selengut J, Paulsen IT, et al. (2003) The complete sequence of the Arabidopsis and tomato pathogen *Pseudomonas syringae* pv. *tomato* DC3000. Proc Natl Acad Sci U S A 100: 10181-10186.
5. Feil H, Feil WS, Chain P, Larimer F, Dibartolo G, et al. (2005) Comparison of the complete genome sequences of *Pseudomonas syringae* pv. *syringae* B728a and pv. *tomato* DC3000. Proc Natl Acad Sci U S A 102: 11064-11069.
6. Taguchi F, Shimizu R, Inagaki Y, Toyoda K, Shiraishi T, et al. (2003) Post-translational modification of flagellin determines the specificity of HR induction. Plant Cell Physiol 44: 342-349.
